# Trends in cardiovascular diseases and heart disease death rates among adults aged 45-64: Brazil, 2000-2017

**DOI:** 10.1590/1516-3180.2019.1373.220719

**Published:** 2019-08-29

**Authors:** Paulo Andrade Lotufo

**Affiliations:** I MD, DrPH. Full Professor, Department of Internal Medicine, Faculdade de Medicina da Universidade de São Paulo (FMUSP), São Paulo (SP), Brazil.

In a previous editorial in this Journal this year, I addressed the question of whether the risk of death due to cancer in Brazil will surpass the risk of death due to cardiovascular diseases at some point over the next decades. The conclusion was that “the risks of death due to cardiovascular diseases, independent of aging, are higher than the numbers and risks relating to cancer, for both sexes.”^1^ This result differed from what was observed recently in the United States.^2^

One criticism of approaches in which all age strata are analyzed is that the older that people are when they die, the more frequent it is that comorbidities will be present, especially coexistence of heart disease and cancer. Consequently, among elderly people, mortality data based on the underlying cause of death do not reflect the primary determinant of death, as occurs in cases of death among young people. A second concern is that the impact of death among middle-aged individuals has much greater social, economic and psychological implications than does death among elderly people.

The differences between rates of occurrence of circulatory diseases and neoplasm among people aged 45 to 64 years for the whole of Brazil from 2000 to 2017 were analyzed using the Ministry of Health mortality databank. The first approach consisted of comparison of proportional mortality; the second step was to determine the annual percentage change (APC) in the mortality rates, calculated using Poisson regression, thereby revealing the inflection points of the mortality rate curves.

Cardiovascular diseases and cancer are the cause of more than half of deaths among adults in Brazil. The excess of deaths for men (67%) was constant over this entire period. Comparison of proportional mortality for each sex between the periods 2000-2002 and 2015-2017 showed that the proportion of deaths due to cardiovascular diseases decreased both for men (from 30.1% to 27.6%) and for women (31.1% to 29.9%), and that the proportion of deaths due to cancer increased both for men (16.6% to 19.9%) and for women (14.4% to 17.9%). The rise in the proportion of or men, the percentage of deaths due to circulatory diseases was 30.1%, and for cancer, 16.6% in 2000-2002 and changed to 27.6% for circulatory disorders

**Figure 1.**
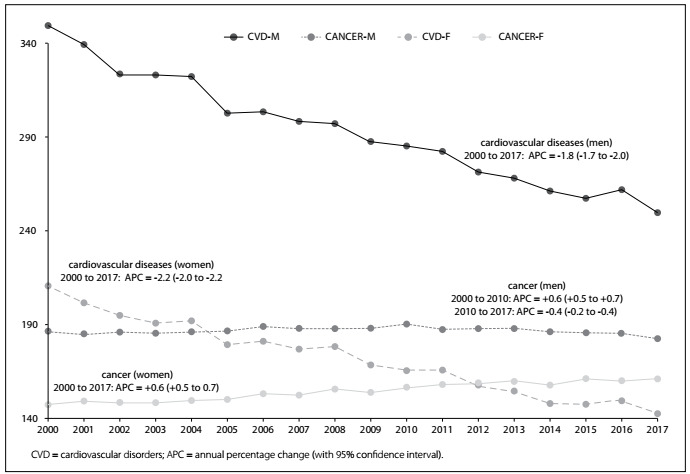
Mortality rates due to cardiovascular diseases and cancer in Brazil from 2000 to 2017, for men and women aged 45 to 64 years.

Figure 1 shows different temporal trends of the mortality rates (per 100,000 inhabitants) due to cardiovascular diseases and cancer for each sex, for people aged 45 to 64 years. For men, the risk of death due to all cardiovascular diseases declined year by year at a relatively constant pace. In contrast, the temporal trends regarding death due to cancer among men had a peak in 2010 and has been declining since then.

For women, there was a shift in the risk of death after 2012. In that year, the rates due to cancer and cardiovascular diseases matched. The reason for the shift was a downward trend regarding cardiovascular diseases and an upward trend regarding neoplasm since 2000.

Table 1 shows the temporal trends for coronary heart diseases (CHD), stroke and other cardiovascular diseases (most of them are myocardiopathy). All of these categories presented significant reductions in their rates over 2000-2017. Despite an initial, lower rate of cardiovascular diseases among women than among men, the annual decline was faster for women.

**Table 1.** Annual percentage change (APC) and 95% confidence interval (95% CI) for death rates due to cardiovascular disorders (CVD) in Brazil from 2000 to 2017, for women and men aged 45 to 64 years

	Women		Men	
	period	APC (95% CI)	period	APC (95% CI)
Coronary heart disease	2000-17	-1.3 (-1.5 to-1.1)	2000-17	-1.1 (-1.3 to -1.0)
Stroke	2000-17	-3.4 (-3.5 to -3.2)	2000-08	-2.9 (-3.7 to -2.1)
Other CVD	2000-17	-1.8 (-2.0 to -1.6)	2000-17	-1.4 (-1.6 to -1.3)

Table 2 shows the patterns of mortality rates due to different types of cancer for women and men, calculated according to the annual percentage change. The most notable difference according to gender was in relation to lung cancer. Women presented an alarming yearly increase in deaths of 2.4%; in contrast, men had an annual reduction of 1.3%. The mortality rates due to upper aerodigestive cancers (mouth, nose, pharynx, larynx and esophagus), which along with lung cancer are closely associated with the smoking habit, climbed for women but declined for men from 2008 onwards. The combination of those diseases did not materially change the trend according to gender.

**Table 2:** Annual percentage change (APC) and 95% confidence interval (95% CI) for death rates due to cancer in Brazil from 2000 to 2017, for women and men aged 45 to 64 years

	Women			
	period	APC (95% CI)	period	APC (95% CI)
Lung		2.4 (2.2 to 2.7)	2000-17	-1.3 (-1.4 to -1.1)
UAD	2000-07	1.5 (0.6 to 2.4)	2000-09	0.2 (-0.1 to 0.5)
	2007-17	-0.6 (-1.0 to -0.1)	2009-17	-0.7 (-1.0 to -0.4)
Lung + UAD	2000-17	1.7 (1.5 to 1.9)	2000-08	-0.2 (-0.5 to 0.0)
			2008-17	-1.0 (-1.2 to -0.8)
Stomach	2000-17	-0.7 (-1.0 to -0.5)	2000-17	-2.0 (-2.1 to -1.8)
Colon + rectum	2000-17	2.1 (1.9 to 2.4)	2000-17	2.6 (2.4 to 2.8)
Liver + biliary tract	2000-017	0.5 (0.3 to 0.7)	2000-12	1.9 (1.4 to 2.5)
			2012-17	-0.4 (-2.0 to 1.3)
Pancreas	2000-17	1.9 (1.6 to 2.2)	2000-17	2.0 (1.8 to 2.2)
CNS	2000-11	1.4 (1.1 to 1.8)	2000-17	0.7 (0.4 to 1.0)
	2011-17	0.2 (-0.6 to 0.9)		
Lymphoma	2000-17	-0.4 (-0.8 to 0.0)	2000-17	-0.7 (-1.1 to -0.3)
Leukemia	2000-17	-0.2 (-0.6 to 0.2)	2000-17	-0.4 (-0.8 to -0.1)
Myeloma	2000-17	0.7 (0.2 to 1.2)	2000-17	1.2 (0.7 to 1.8)
Prostate	N/A		2000-17	-0.2 (0.3 to 0.0)
Breast	2000-17	0.8 (0.7 to 0.9)		
Ovarium	2000-17	0.9 (0.7 to 1.2)		
Endometrium	2000-09	-2.7 (-3.5 to -1.8)		
	2009-17	0.2 (-0.8 to 1.2)		
Uterine cervix	2000-14	-1.5 (-1.7 to -1.3)		
	2014-17	1.8 (-0.5 to 4.1)		

UAD = upper aerodigestive neoplasm (in mouth, nose, pharynx, larynx and esophagus); CNS = central nervous system.

Cancers of the colon, rectum, liver, biliary tract and pancreas, myelomas and neoplasms of the central nervous system showed annual increases for both sexes (except myelomas, only for women). Declines in rates were observed for stomach cancer for both genders, and for leukemias and lymphomas for men. The risk of death due to prostate cancer did not change significantly. Breast and ovarium cancer showed an upward trend, but deaths due to endometrial and uterine cervical cancer showed a downward trend.

This description of the mortality rates among middle-aged women and men in Brazil in this century reveals some crucial issues relating to medical and public health:

1. The excess of premature male deaths due to cardiovascular diseases and cancer was constant, with a male-to-female ratio of 2:1.
2. The reduction in cardiovascular diseases among middle-aged men and women was impressive, and this means that the influence of weight gain was not still observed. The death rate due to diabetes also declined (data not shown).
3. The risk of death due to cancer among women surpassed the risk due to circulatory diseases after 2012.
4. The impact of smoking-associated cancer differed according to gender. It can be speculated that, over the next five to ten years, the number of deaths due to lung cancer will be higher among women.
5. The death rates due to neoplasms located in the colon, rectum, liver, biliary tract and pancreas are rising steadily for both sexes. In contrast, stomach cancer deaths are declining.
6. Occurrences of neoplasms located in the central nervous system are rising, as also are occurrences of leukemias, lymphomas and myelomas.
7. Despite successive annual campaigns against breast and prostate cancer, no impact can be seen. The prostate cancer rate is flatlining and the breast cancer rate is increasing.

Full comprehension of these patterns will require more analysis, including to verify the patterns of mortality due to other noncommunicable diseases such as chronic obstructive pulmonary disease, hepatic cirrhosis and chronic kidney failure. Moreover, the impact of external causes of deaths among men as competitive risks needs to be further addressed.
